# Self-Propulsion of Two Contacting Bubbles Due to the Radiation Interaction Force

**DOI:** 10.3390/mi14081615

**Published:** 2023-08-16

**Authors:** Alexander A. Doinikov, Thomas Micol, Cyril Mauger, Philippe Blanc-Benon, Claude Inserra

**Affiliations:** 1 University Lyon, École Centrale de Lyon, INSA de Lyon, CNRS, LMFA UMR 5509, F-69002 Lyon, France; 2 University Lyon, Université Claude Bernard Lyon 1, Centre Léon Bérard, INSERM, UMR 1032, LabTAU, F-69003 Lyon, France

**Keywords:** gas bubble, radiation interaction force, viscous liquid, self-propulsion, acoustic manipulation

## Abstract

In this paper, we consider a new bubble-based microswimmer composed of two contacting bubbles. Under the action of an acoustic field, both bubbles are oscillating, and locomotion of the two-bubble system is observed. A theory is developed that allows one to calculate the acoustic radiation interaction forces between two gas bubbles in an incompressible viscous liquid for any small separation distance between the bubbles. This theory is used to demonstrate that two acoustically excited bubbles can create a self-propelled microswimmer due to a nonzero net force experienced by the bubbles when they come in contact. Experimental evidence of the creation of such a swimmer and of its motion is provided.

## 1. Introduction

Interest in artificial self-propelled microswimmers, which could be used in microfluidic and biomedical applications, has existed for decades. The range of possible applications of microswimmers is very wide: cargo transport, micromixing, sensing, targeted drug delivery, indirect manipulation of cells, and other microscopic objects, such as microsurgery, etc.

Different types of microswimmers have been proposed [1], which are actuated by various external energy sources such as light [2], electric [3], magnetic [4] or acoustic [5] fields. One of the promising ways is the development of acoustically controlled bubble-based microswimmers [6,7,8], which have attracted great attention due to their non-invasiveness and cheap implementation. Acoustically-driven swimmers have been used to capture and move micro-objects [9], to mix fluids [10], and to drive the assembly of microparticles, cells, or microstructures [11]. Bubble-based swimmers are usually composed of a rigid microcapsule that contains an air bubble inside [6,7]. Upon the application of an acoustic field, periodic oscillations of the bubble are induced, resulting in the motion of the capsule. Publications devoted to these investigations commonly attribute the source of locomotion to acoustic microstreaming generated by the bubble. With the aim of accurately manipulating and controlling acoustically-powered swimmers, microrobots composed of two bubbles have been recently designed [8,12]. In addition to the translational motion of the robot, a rotational motion is observed that results from the asymmetry in the bubble oscillations amplitudes and the induced microstreaming. Such asymmetry is generated by switching the frequency of the driving acoustic field between the resonant frequencies of each of the two bubbles. For a two-bubble robot composed of bubbles with equilibrium radii of about 45 µm and driven at a frequency of 40 kHz [8], it has been shown that the translational velocity can reach 8 mm/s, which corresponds to almost 50 body lengths per second. The predominant propulsion mechanism was established to be the bubble-induced microstreaming, while the radiation pressure exerted by the acoustic field on the swimmer was shown to be negligible.

In this paper, we propose a new acoustically powered system composed of two contacting bubbles driven by an acoustic field. We demonstrate that two acoustically excited contacting bubbles can create a self-propelled microswimmer due to the radiation interaction force acting between the bubbles. It is worth noting that a similar geometry has been considered by Pak et al. [13] in relation to two contacting rigid spheres that rotate about their axis of symmetry. This structure was called “snowman” by the authors. In our paper, we will refer to the robot based on two contacting bubbles as a “bubbleman”.

When two bubbles are subjected to an acoustic field, the scattered wave generated by one bubble produces a time-averaged force on the other bubble, and vice versa. These forces make the bubbles attract or repel each other. This effect was first reported by C. A. Bjerknes and his son V. F. K. Bjerknes [14], and since then it has been well known in acoustics. In modern literature, this force is referred to by several names: radiation interaction force, secondary radiation force, Bjerknes force, or secondary Bjerknes force [15,16]. C. A. Bjerknes has derived an analytical expression for the time-averaged interaction force between two bubbles, assuming that the bubbles pulsate radially in an incompressible nonviscous liquid and that the separation distance between the bubbles is large compared to the bubble radii. Under such conditions, the system of two interacting bubbles is conservative, which means that the forces on the bubbles are equal and opposite. Doinikov [17,18] has shown that in a viscous liquid, the interaction forces experienced by two bubbles are no longer equal and opposite as the liquid viscosity breaks the conservatism of the system of two interacting bubbles. However, the assumption that the separation distance between the bubbles is large compared to the bubble radii, which was used in his derivation, does not allow one to apply his results to bubbles in contact.

In the present paper, a theory is developed that allows one to calculate the radiation interaction forces between two bubbles in an incompressible viscous liquid for any small separation distance between the bubbles. This theory reveals that if the bubbles come in contact, their agglomerate experiences a nonzero net force, which causes locomotion of the bubbleman. We also provide experimental evidence of this effect.

## 2. Theory

### 2.1. Interaction Force between Two Bubbles in an Acoustic Field

**Figure 1:**
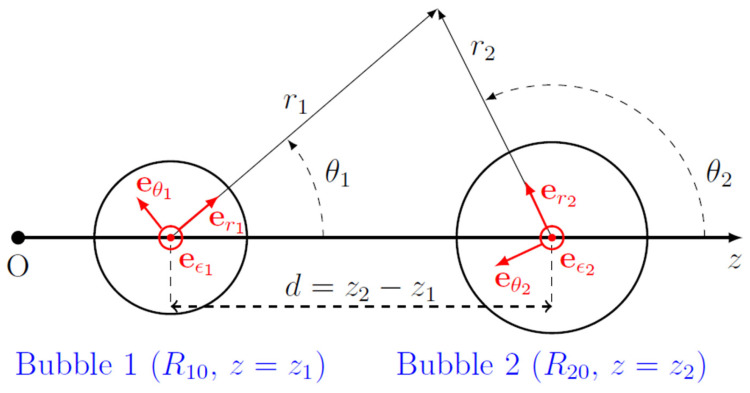
Coordinate systems used in calculations.

We begin with the calculation of the radiation interaction force, also referred to as the secondary radiation force, which acts between two spatially separated gas bubbles in an acoustically excited liquid. This calculation is based on the theory developed by Doinikov et al. [19] for acoustic microstreaming generated by two interacting bubbles. The bubbles are assumed to be immersed in a viscous, incompressible liquid and undergo axisymmetric oscillations. We used two spherical coordinate systems, $\left(r_{1},\theta_{1},\varepsilon_{1}\right)$ and $\left(r_{2},\theta_{2},\varepsilon_{2}\right)$, which originate at the equilibrium centers of bubbles 1 and 2, respectively, and in which the direction $\theta_{1}=\theta_{2}=0$ corresponds to the *z* axis; see Figure 1. The distance between the equilibrium centers of the bubbles is denoted by *d*.

We assume that each bubble can undergo several oscillation modes, which can have different frequencies because some of the modes can be excited parametrically. By definition, the radiation interaction force is a time-averaged quantity. Since time averaging annihilates terms that arise from the interaction of modes with different frequencies, a contribution to the force can only come from the interaction of modes with the same frequency. Therefore, the modes can be divided into groups in accordance with their frequencies and the contributions of such groups can be considered independently.

Let us assume that the *j*th bubble (*j* = 1,2) undergoes $N_{j}$ modes with a frequency ω. Let these modes have numbers $M_{1}^{\left(j\right)}$, $M_{2}^{\left(j\right)}$,…, $M_{N_{j}}^{\left(j\right)}$. Then, the perturbation of the bubble surface produced by these modes can be represented by

(1) $$r_{s}^{\left(j\right)}=R_{j0}+e^{-i\omega t}\sum_{n=M_{1}^{\left(j\right)}}^{M_{N_{j}}^{\left(j\right)}}s_{n}^{\left(j\right)}P_{n}(\mu_{j}),$$

where $r_{s}^{\left(j\right)}$ is the radial coordinate of the surface of the *j*th bubble, $R_{j0}$ is the equilibrium radius of the *j*th bubble, $P_{n}$ is the Legendre polynomial of degree *n*, $\mu_{j}=\cos\theta_{j}$, and $s_{n}^{\left(j\right)}$ is the complex amplitude of the *n*th mode of the *j*th bubble, which is assumed to be known, measured experimentally [19], or evaluated theoretically [20,21].

The time-averaged acoustic radiation force on the *j*th bubble can be represented as [22]

(2) $$F_{j}=\int_{S_{j0}}\left\{-\rho \langle (n_{j}\cdot v)v\rangle +2\eta (n_{j}\cdot \nabla )v_{E}+\eta n_{j}\times \left(\nabla \times v_{E}\right)-p_{E}n_{j}\right\}dS_{j0},$$

where $S_{j0}$ is the surface of the *j*th bubble at rest, $n_{j}$ is the unit outward normal to $S_{j0}$, ρ is the liquid density, v is the first-order liquid velocity generated by the bubbles, η is the dynamic liquid viscosity, $v_{E}$ is the Eulerian velocity of acoustic streaming, $p_{E}$ is the time-averaged pressure corresponding to $v_{E}$, and 〈〉 means the time average.

The total first-order liquid velocity generated by both bubbles is given by

(3) $$v=v^{\left(1\right)}+v^{\left(2\right)},$$

where $v^{\left(j\right)}$ is the first-order liquid velocity generated by the *j*th bubble. The components of $v^{\left(j\right)}$ are shown in [19] to be calculated by

(4) $$v_{r}^{\left(j\right)}(r_{j},\theta_{j})=-\frac{e^{-i\omega t}}{r_{j}}\sum_{n=0}^{\infty }\left(n+1)\left[a_{n}^{\left(j\right)}{\left(\frac{R_{j0}}{r_{j}}\right)}^{n+1}+b_{n}^{\left(j\right)}nh_{n}^{\left(1\right)}(k_{v}r_{j})\right]P_{n}(\mu_{j}\right),$$

(5) $$v_{\theta }^{\left(j\right)}(r_{j},\theta_{j})=\frac{e^{-i\omega t}}{r_{j}}\sum_{n=1}^{\infty }\left\{a_{n}^{\left(j\right)}{\left(\frac{R_{j0}}{r_{j}}\right)}^{n+1}-b_{n}^{\left(j\right)}\left[h_{n}^{\left(1\right)}(k_{v}r_{j})+k_{v}r_{j}h_{n}^{(1)/}(k_{v}r_{j})\right]\right\}P_{n}^{1}(\mu_{j}),$$

where $h_{n}^{\left(1\right)}$ is the spherical Hankel function of the first kind, $k_{v}=\left(1+i\right)/\delta$ is the viscous wavenumber, $\delta =\sqrt{2\nu /\omega }$ is the viscous penetration depth, ν=η/ρ is the kinematic liquid viscosity, the prime denotes the derivative with respect to the argument in brackets (such as the term $h_{n}^{(1)/}(k_{v}r_{j})\;$ in Equation (5)), and $P_{n}^{1}$ is the associated Legendre polynomial of the first order and degree *n*. Expressions for the constants $a_{n}^{\left(j\right)}$ and $b_{n}^{\left(j\right)}$, called linear scattering coefficients, are provided in Appendix A of [19].

Equations (4) and (5) give the components of $v^{\left(j\right)}$ in the coordinates of the *j*th bubble. However, to make use of Equation (2), we also need to know how these components are expressed in the coordinates of the other bubble, i.e., how the components of $v^{\left(1\right)}$ are expressed in the coordinates $\left(r_{2},\theta_{2}\right)$ of bubble 2 and vice versa. Necessary equations are provided in Appendix A of [19]; see Equations (A11), (A13), (A15) and (A16) therein. By using these equations, the components of the total first-order liquid velocity are represented by

(6) $$v_{r}(r_{j},\theta_{j})=e^{-i\omega t}\sum_{n=0}^{\infty }V_{rn}^{\left(j\right)}(r_{j})P_{n}(\mu_{j}),$$

(7) $$v_{\theta }(r_{j},\theta_{j})=e^{-i\omega t}\sum_{n=1}^{\infty }V_{\theta n}^{\left(j\right)}(r_{j})P_{n}^{1}(\mu_{j}),$$

where $V_{rn}^{\left(j\right)}(r_{j})$ and $V_{\theta n}^{\left(j\right)}(r_{j})$ are calculated by

(8) $$V_{rn}^{\left(1\right)}(r_{1})=-\frac{n+1}{r_{1}}\left[a_{n}^{\left(1\right)}{\left(\frac{R_{10}}{r_{1}}\right)}^{n+1}+b_{n}^{\left(1\right)}nh_{n}^{\left(1\right)}(k_{v}r_{1})\right]+\frac{n}{r_{1}}{\left(\frac{r_{1}}{d}\right)}^{n}\sum_{m=0}^{\infty }{\left(-1\right)}^{m}C_{nm}\xi_{2}^{m+1}a_{m}^{\left(2\right)}\;-(2n+1)i^{n}\sqrt{n(n+1)}\frac{j_{n}(k_{v}r_{1})}{r_{1}}\sum_{m=1}^{\infty }\frac{\sqrt{m(m+1)}}{(2m+1)i^{m}}b_{m}^{\left(2\right)}\sum_{l=0}^{\infty }i^{-l}(2l+1)C_{n0l0}^{m0}C_{n1l0}^{m1}h_{l}^{\left(1\right)}(k_{v}d),$$

(9) $$V_{rn}^{\left(2\right)}(r_{2})=-\frac{n+1}{r_{2}}\left[a_{n}^{\left(2\right)}{\left(\frac{R_{20}}{r_{2}}\right)}^{n+1}+b_{n}^{\left(2\right)}nh_{n}^{\left(1\right)}(k_{v}r_{2})\right]+\frac{{\left(-1\right)}^{n}n}{r_{2}}{\left(\frac{r_{2}}{d}\right)}^{n}\sum_{m=0}^{\infty }C_{nm}\xi_{1}^{m+1}a_{m}^{\left(1\right)}\;-(2n+1)i^{n}\sqrt{n(n+1)}\frac{j_{n}(k_{v}r_{2})}{r_{2}}\sum_{m=1}^{\infty }\frac{\sqrt{m(m+1)}}{(2m+1)i^{m}}b_{m}^{\left(1\right)}\sum_{l=0}^{\infty }{\left(-1\right)}^{l}i^{-l}(2l+1)C_{n0l0}^{m0}C_{n1l0}^{m1}h_{l}^{\left(1\right)}(k_{v}d),$$

(10) $$V_{\theta n}^{\left(1\right)}(r_{1})=\frac{1}{r_{1}}\left\{a_{n}^{\left(1\right)}{\left(\frac{R_{10}}{r_{1}}\right)}^{n+1}-b_{n}^{\left(1\right)}\left[h_{n}^{\left(1\right)}(k_{v}r_{1})+k_{v}r_{1}h_{n}^{(1)/}(k_{v}r_{1})\right]\right\}+\frac{1}{r_{1}}{\left(\frac{r_{1}}{d}\right)}^{n}\sum_{m=0}^{\infty }{\left(-1\right)}^{m}C_{nm}\xi_{2}^{m+1}a_{m}^{\left(2\right)}\;-\frac{(2n+1)i^{n}}{\sqrt{n(n+1)}}\frac{j_{n}(k_{v}r_{1})+k_{v}r_{1}j_{n}^{/}(k_{v}r_{1})}{r_{1}}\sum_{m=1}^{\infty }\frac{\sqrt{m(m+1)}}{(2m+1)i^{m}}b_{m}^{\left(2\right)}\sum_{l=0}^{\infty }i^{-l}(2l+1)C_{n0l0}^{m0}C_{n1l0}^{m1}h_{l}^{\left(1\right)}(k_{v}d),$$

(11) $$V_{\theta n}^{\left(2\right)}(r_{2})=\frac{1}{r_{2}}\left\{a_{n}^{\left(2\right)}{\left(\frac{R_{20}}{r_{2}}\right)}^{n+1}-b_{n}^{\left(2\right)}\left[h_{n}^{\left(1\right)}(k_{v}r_{2})+k_{v}r_{2}h_{n}^{(1)/}(k_{v}r_{2})\right]\right\}+\frac{{\left(-1\right)}^{n}}{r_{2}}{\left(\frac{r_{2}}{d}\right)}^{n}\sum_{m=0}^{\infty }C_{nm}\xi_{1}^{m+1}a_{m}^{\left(1\right)}\;-\frac{(2n+1)i^{n}}{\sqrt{n(n+1)}}\frac{j_{n}(k_{v}r_{2})+k_{v}r_{2}j_{n}^{/}(k_{v}r_{2})}{r_{2}}\sum_{m=1}^{\infty }\frac{\sqrt{m(m+1)}}{(2m+1)i^{m}}b_{m}^{\left(1\right)}\sum_{l=0}^{\infty }{\left(-1\right)}^{l}i^{-l}(2l+1)C_{n0l0}^{m0}C_{n1l0}^{m1}h_{l}^{\left(1\right)}(k_{v}d).$$

Here, $C_{nm}=(n+m)!/\left(n!m!\right)$, $\xi_{j}=R_{j0}/d$, $j_{n}$ is the spherical Bessel function, and $C_{l_{1}m_{1}l_{2}m_{2}}^{LM}$ are the Clebsch–Gordan coefficients [23,24,25]. The superscript (j) in $V_{rn}^{\left(j\right)}$ and $V_{\theta n}^{\left(j\right)}$ emphasizes that these quantities are taken in the coordinates of the *j*th bubble. According to the theory developed in [19], Equations (8)–(11) are valid for $r_{j}<d$. This is sufficient for our purpose because we will see below that the expression for the interaction force only contains the values of $V_{rn}^{\left(j\right)}$ and $V_{\theta n}^{\left(j\right)}$ at $r_{j}=R_{j0}$.

Equations (6) and (7) allow us to calculate the first term in the integrand of Equation (2). To calculate the other terms, we again use the results of [19].

According to Equations (2.17) and (2.18) of [19], the components of the Eulerian streaming velocity $v_{E}$ are given by

(12) $$v_{Er}(r_{j},\theta_{j})=-\frac{1}{r_{j}}\sum_{n=1}^{\infty }n(n+1)\Psi_{n}^{\left(j\right)}(r_{j})P_{n}(\mu_{j}),$$

(13) $$v_{E\theta }(r_{j},\theta_{j})=-\frac{1}{r_{j}}\sum_{n=1}^{\infty }\left[\Psi_{n}^{\left(j\right)}(r_{j})+r_{j}\Psi_{n}^{(j)/}(r_{j})\right]P_{n}^{1}(\mu_{j}),$$

where $\Psi_{n}^{\left(j\right)}(r_{j})$ and $\Psi_{n}^{(j)/}(r_{j})$ are calculated by Equations (C8) and (C17) of [19]. Equations (12) and (13) allow us to calculate the terms of Equation (2) that depend on $v_{E}$ and $p_{E}$.

With the help of Equations (6), (7) and (A1)–(A4) from Appendix A of the present paper, the first term in the integrand of Equation (2) brings

T1(j)=−∫Sj0ρ〈(nj⋅v)v〉dSj0=−πρRj02ezRe∫0πvr∗(vrcosθj−vθsinθj)sinθjdθj

=−πρRj02ezRe{∑n,m=0∞Vrn(j)(Rj0)Vrm(j)∗(Rj0)∫−11Pn(μj)Pm(μj)μjdμj

$$-\sum_{\begin{array}{l} n=1\\ m=0 \end{array}}^{\infty }V_{\theta n}^{\left(j\right)}(R_{j0})V_{rm}^{(j)*}(R_{j0})\int_{-1}^{1}P_{n}^{1}(\mu_{j})P_{m}(\mu_{j})\sqrt{1-\mu_{j}^{2}}d\mu_{j}\}$$

=−2πρRj02ezRe{Vr0(j)(Rj0)Vr1(j)∗(Rj0)+∑n=1∞1(2n+1)2{Vrn(j)(Rj0)[nVr(n−1)(j)∗(Rj0)+(n+1)Vr(n+1)(j)∗(Rj0)]

(14) $$-n(n+1)V_{\theta n}^{\left(j\right)}(R_{j0})\left[V_{r(n+1)}^{(j)*}(R_{j0})-V_{r(n-1)}^{(j)*}(R_{j0})\right]\}\}.$$

The contribution of the second term is calculated as follows:

$$T_{2}^{\left(j\right)}=2\eta \int_{S_{j0}}(n_{j}\cdot \nabla )v_{E}dS_{j0}=4\pi \eta R_{j0}^{2}e_{z}\int_{-1}^{1}{\left(\frac{\partial v_{Er}}{\partial r_{j}}\mu_{j}-\frac{\partial v_{E\theta }}{\partial r_{j}}\sqrt{1-\mu_{j}^{2}}\right)}_{r_{j}=R_{j0}}d\mu_{j}$$

$$=4\pi \eta R_{j0}^{2}e_{z}\{-\sum_{n=1}^{\infty }n(n+1)\left[\frac{\Psi_{n}^{(j)/}(R_{j0})}{R_{j0}}-\frac{\Psi_{n}^{\left(j\right)}(R_{j0})}{R_{j0}^{2}}\right]\int_{-1}^{1}P_{n}(\mu_{j})\mu_{j}d\mu_{j}$$

$$-\sum_{n=1}^{\infty }\left[\frac{\Psi_{n}^{\left(j\right)}(R_{j0})}{R_{j0}^{2}}-\frac{\Psi_{n}^{(j)/}(R_{j0})}{R_{j0}}-\Psi_{n}^{(j)//}(R_{j0})\right]\int_{-1}^{1}P_{n}^{1}(\mu_{j})\sqrt{1-\mu_{j}^{2}}d\mu_{j}\}$$

(15) $$=\frac{16}{3}\pi \eta R_{j0}^{2}e_{z}\left[\frac{2\Psi_{1}^{\left(j\right)}(R_{j0})}{R_{j0}^{2}}-\frac{2\Psi_{1}^{(j)/}(R_{j0})}{R_{j0}}-\Psi_{1}^{(j)//}(R_{j0})\right].$$

Making use of the equation

(16) $$n_{j}\times \left(\nabla \times v_{E}\right)=\frac{e_{\theta j}}{r_{j}}[\frac{\partial v_{Er}}{\partial \theta_{j}}-\frac{\partial (r_{j}v_{E\theta })}{\partial r_{j}}],$$

the calculation of the third term results in

$$T_{3}^{\left(j\right)}=\eta \int_{S_{j0}}n_{j}\times \left(\nabla \times v_{E}\right)dS_{j0}$$

$$=2\pi \eta R_{j0}e_{z}\sum_{n=1}^{\infty }\left[\frac{n(n+1)}{R_{j0}}\Psi_{n}^{\left(j\right)}(R_{j0})-2\Psi_{n}^{(j)/}(R_{j0})-R_{j0}\Psi_{n}^{(j)//}(R_{j0})\right]\int_{-1}^{1}P_{n}^{1}(\mu_{j})\sqrt{1-\mu_{j}^{2}}d\mu_{j}$$

(17) $$=-\frac{8}{3}\pi \eta R_{j0}^{2}e_{z}\left[\frac{2\Psi_{1}^{\left(j\right)}(R_{j0})}{R_{j0}^{2}}-\frac{2\Psi_{1}^{(j)/}(R_{j0})}{R_{j0}}-\Psi_{1}^{(j)//}(R_{j0})\right].$$

To calculate the fourth term of Equation (2), we first need to calculate $p_{E}$. In order to perform this calculation, we only need the expression of $p_{E}$ at $r_{j}=R_{j0}$. In addition, it is reasonable to assume that the time-averaged gas pressure inside the *j*th bubble has the same value at all points of the gas medium, which means that $p_{E}$ should be constant, independent of $\theta_{j}$, at $r_{j}=R_{j0}$. It is easy to check that, in this case, the fourth term of Equation (2) does not contribute to the force.

To calculate $\Psi_{1}^{\left(j\right)}(R_{j0})$, $\Psi_{1}^{(j)/}(R_{j0})$ and $\Psi_{1}^{(j)//}(R_{j0})$, we use Equations (C8), (C17) and (C18) of [19],

(18) $$\Psi_{1}^{\left(j\right)}(R_{j0})=C_{110}^{\left(j\right)}+\frac{C_{210}^{\left(j\right)}}{R_{j0}^{2}}+R_{j0}C_{310}^{\left(j\right)}+R_{j0}^{3}C_{410}^{\left(j\right)},$$

(19) $$\Psi_{1}^{(j)/}(R_{j0})=-\frac{2C_{210}^{\left(j\right)}}{R_{j0}^{3}}+C_{310}^{\left(j\right)}+3R_{j0}^{2}C_{410}^{\left(j\right)},$$

(20) Ψ1(j)//(Rj0)=6C210(j)Rj04+6RCj0410(j),

where the constants $C_{110}^{\left(j\right)}$–$C_{410}^{\left(j\right)}$ are calculated by Equations (C30)–(C33) of [19].

With the help of Equations (18)–(20), one obtains

(21) $$\frac{\Psi_{1}^{\left(j\right)}(R_{j0})}{R_{j0}^{2}}-\frac{\Psi_{1}^{(j)/}(R_{j0})}{R_{j0}}-\frac{\Psi_{1}^{(j)//}(R_{j0})}{2}=\frac{C_{110}^{\left(j\right)}}{R_{j0}^{2}}-5R_{j0}C_{410}^{\left(j\right)}.$$

Combining Equations (14), (15), (17) and (21) brings the following expression for the radiation interaction force on the *j*th bubble:

Fj=16π3η(C110(j)−5Rj03C410(j))−2πρRj02Re{Vr0(j)(Rj0)Vr1(j)∗(Rj0)+∑n=1∞1(2n+1)2{Vrn(j)(Rj0)[nVr(n−1)(j)∗(Rj0)+(n+1)Vr(n+1)(j)∗(Rj0)]

(22) $$-n(n+1)V_{\theta n}^{\left(j\right)}(R_{j0})\left[V_{r(n+1)}^{(j)*}(R_{j0})-V_{r(n-1)}^{(j)*}(R_{j0})\right]\}\},$$

where $C_{110}^{\left(j\right)}$ and $C_{410}^{\left(j\right)}$ are constants that are calculated by Equations (C30) and (C33) of [19], Re means “the real part of”, and the asterisk denotes complex conjugate. Note that the force is directed along the line joining the equilibrium centers of the bubbles, i.e., along the *z* axis in Figure 1. Therefore, $F_{j}>0$ means that the force acts in the positive direction of the *z* axis, while $F_{j}<0$ means that the force acts in the negative direction of the *z* axis.

In this subsection we have derived an analytical formula that allows one to calculate the interaction forces on two bubbles in the case that the bubbles undergo strong shape modes, which are excited parametrically, and for which we know the magnitudes and the phases of these modes.

### 2.2. Linear Scattering Coefficients When Parametric Excitation Is Absent

If the parametric excitation of shape modes is absent, $a_{n}^{\left(j\right)}$ and $b_{n}^{\left(j\right)}$ can be expressed in terms of the amplitude of the imposed acoustic pressure. In this case, Equation (1) is transformed to

(23) $$r_{s}^{\left(j\right)}=R_{j0}+e^{-i\omega t}\sum_{n=0}^{\infty }s_{n}^{\left(j\right)}P_{n}(\mu_{j}),$$

The linearized equations of an incompressible viscous liquid are given by [26]

(24) ∇⋅v=0,

(25) $$\frac{\partial v}{\partial t}=-\frac{1}{\rho }\nabla p+\nu \Delta v,$$

where p is the first-order liquid pressure.

In the case of two bubbles, a solution for v is sought as

(26) $$v=v^{\left(1\right)}+v^{\left(2\right)},$$

where $v^{\left(j\right)}$ is represented by

(27) $$v^{\left(j\right)}=\nabla \varphi^{\left(j\right)}+\nabla \times \psi^{\left(j\right)},$$

with the scalar, $\varphi^{\left(j\right)}$, and the vector, $\psi^{\left(j\right)}$, velocity potentials defined by [27]

(28) $$\varphi^{\left(j\right)}=e^{-i\omega t}\sum_{n=0}^{\infty }a_{n}^{\left(j\right)}{\left(\frac{R_{j0}}{r_{j}}\right)}^{n+1}P_{n}(\mu_{j}),$$

(29) $$\psi^{\left(j\right)}=e^{-i\omega t}\psi^{\left(j\right)}(r_{j},\theta_{j})e_{\varepsilon j}=e^{-i\omega t}e_{\varepsilon j}\sum_{n=1}^{\infty }b_{n}^{\left(j\right)}h_{n}^{\left(1\right)}(k_{v}r_{j})P_{n}^{1}(\mu_{j}),$$

where $e_{\varepsilon j}$ is the unit azimuth vector of the *j*th bubble. Note that axial symmetry allows us to set $\varepsilon_{1}=\varepsilon_{2}$ and $e_{\varepsilon 1}=e_{\varepsilon 2}$.

Substituting Equation (27) into Equation (25) and taking into account that $\psi^{\left(j\right)}$ obeys the equation $(\Delta +k_{v}^{2})\psi^{\left(j\right)}=0$ [27], one finds that the first-order scattered pressure generated by the *j*th bubble is given by

(30) $$p^{\left(j\right)}=i\omega \rho \varphi^{\left(j\right)}.$$

Note that the total first-order scattered pressure is equal to $p=p^{\left(1\right)}+p^{\left(2\right)}$.

We use the boundary condition for normal stress at the surface of the *j*th bubble,

(31) $$P_{gj}{\left(\frac{V_{j0}}{V_{j}(t)}\right)}^{\gamma }={p|}_{r_{j}=R_{j0}}-2\eta {\frac{\partial v_{r}(r_{j},\theta_{j})}{\partial r_{j}}|}_{r_{j}=R_{j0}}+p_{st}^{\left(j\right)}+P_{ac}(t)+P_{0},$$

where the gas pressure within the bubbles is assumed to be spatially homogeneous and to obey the adiabatic law, $P_{gj}$ is the equilibrium gas pressure inside the *j*th bubble, $V_{j0}=(4/3)\pi R_{j0}^{3}$ is the equilibrium volume of the *j*th bubble, $V_{j}(t)$ is the instantaneous volume of the *j*th bubble, γ is the specific hear ratio of the gas, $P_{0}$ is the hydrostatic pressure in the liquid, $P_{ac}(t)=P_{a}e^{-i\omega t}$ is the imposed acoustic pressure, and $p_{st}^{\left(j\right)}$ is the pressure of surface tension on the surface of the *j*th bubble, given by [28]

(32) $$p_{st}^{\left(j\right)}=\frac{2\sigma }{R_{j0}}+e^{-i\omega t}\frac{\sigma }{R_{j0}^{2}}\sum_{n=0}^{\infty }\left(n-1)(n+2)s_{n}^{\left(j\right)}P_{n}(\mu_{j}\right),$$

where σ is the surface tension coefficient.

Accurate to first-order terms, $V_{j}(t)$ is calculated by

(33) $$V_{j}(t)=\int_{0}^{r_{s}^{\left(j\right)}}r^{2}dr\int_{0}^{\pi }\sin\theta_{j}d\theta_{j}\int_{0}^{2\pi }d\varepsilon_{j}=\frac{2\pi }{3}\int_{-1}^{1}{\left(r_{s}^{\left(j\right)}\right)}^{3}d\mu_{j}\approx V_{j0}(1+e^{-i\omega t}\frac{3s_{0}^{\left(j\right)}}{R_{j0}}).$$

To apply Equation (31) and thus calculate $a_{n}^{\left(j\right)}$ and $b_{n}^{\left(j\right)}$, we need to know $\varphi^{\left(1\right)}$ and $v_{r}^{\left(1\right)}$ in the coordinates ($r_{2}$, $\theta_{2}$) and $\varphi^{\left(2\right)}$ and $v_{r}^{\left(2\right)}$ in the coordinates ($r_{1}$, $\theta_{1}$). To this end, we use Equations (A5), (A6), (A11) and (A13) of [19], which give

(34) $$\varphi^{\left(1\right)}(r_{2},\theta_{2})=e^{-i\omega t}\sum_{n,m=0}^{\infty }a_{n}^{\left(1\right)}\xi_{1}^{n+1}{\left(-1\right)}^{m}C_{nm}{\left(\frac{r_{2}}{d}\right)}^{m}P_{m}(\mu_{2}),$$

$$v_{r}^{\left(1\right)}(r_{2},\theta_{2})=e^{-i\omega t}\sum_{n,m=0}^{\infty }P_{m}(\mu_{2})\{\frac{{\left(-1\right)}^{m}m}{d}C_{nm}\xi_{1}^{n+1}a_{n}^{\left(1\right)}{\left(\frac{r_{2}}{d}\right)}^{m-1}$$

(35) $$-\frac{j_{m}(k_{v}r_{2})}{r_{2}}\frac{\sqrt{n(n+1)}}{(2n+1)i^{n}}(2m+1)i^{m}\sqrt{m(m+1)}b_{n}^{\left(1\right)}\sum_{l=0}^{\infty }{\left(-1\right)}^{l}i^{-l}(2l+1)C_{m0l0}^{n0}C_{m1l0}^{n1}h_{l}^{\left(1\right)}(k_{v}d)\},$$

(36) $$\varphi^{\left(2\right)}(r_{1},\theta_{1})=e^{-i\omega t}\sum_{n,m=0}^{\infty }{\left(-1\right)}^{n}a_{n}^{\left(2\right)}\xi_{2}^{n+1}C_{nm}{\left(\frac{r_{1}}{d}\right)}^{m}P_{m}(\mu_{1}),$$

$$v_{r}^{\left(2\right)}(r_{1},\theta_{1})=e^{-i\omega t}\sum_{n,m=0}^{\infty }P_{m}(\mu_{1})\{\frac{{\left(-1\right)}^{n}m}{d}C_{nm}\xi_{2}^{n+1}a_{n}^{\left(2\right)}{\left(\frac{r_{1}}{d}\right)}^{m-1}$$

(37) $$-\frac{j_{m}(k_{v}r_{1})}{r_{1}}\frac{\sqrt{n(n+1)}}{(2n+1)i^{n}}(2m+1)i^{m}\sqrt{m(m+1)}b_{n}^{\left(2\right)}\sum_{l=0}^{\infty }i^{-l}(2l+1)C_{m0l0}^{n0}C_{m1l0}^{n1}h_{l}^{\left(1\right)}(k_{v}d)\}.$$

With the help of Equations (30) and (32)–(37), applying Equation (31) at *j* = 1 and *j* = 2, one obtains for n=0,

(38) $$s_{0}^{\left(1\right)}=\frac{\omega }{\omega_{10}^{2}R_{10}}[(2\tau_{1}-i)a_{0}^{\left(1\right)}-i\sum_{m=0}^{\infty }{\left(-1\right)}^{m}\xi_{2}^{m+1}a_{m}^{\left(2\right)}-\frac{P_{a}}{\rho \omega }],$$

(39) $$s_{0}^{\left(2\right)}=\frac{\omega }{\omega_{20}^{2}R_{20}}\left[(2\tau_{2}-i)a_{0}^{\left(2\right)}-i\sum_{m=0}^{\infty }\xi_{1}^{m+1}a_{m}^{\left(1\right)}-\frac{P_{a}}{\rho \omega }\right],$$

for n=1,

$$(6\tau_{1}-i)a_{1}^{\left(1\right)}+2\tau_{1}[h_{1}^{\left(1\right)}(x_{1})-x_{1}h_{1}^{(1)/}(x_{1})]b_{1}^{\left(1\right)}$$

(40) $$-i\xi_{1}\sum_{m=0}^{\infty }{\left(-1\right)}^{m}(m+1)\xi_{2}^{m+1}a_{m}^{\left(2\right)}+3\sqrt{2}i\tau_{1}[j_{1}(x_{1})-x_{1}j_{1}^{/}(x_{1})]\sum_{m=1}^{\infty }\kappa_{1m}^{\left(2\right)}b_{m}^{\left(2\right)}=0,$$

$$(6\tau_{2}-i)a_{1}^{\left(2\right)}+2\tau_{2}[h_{1}^{\left(1\right)}(x_{2})-x_{2}h_{1}^{(1)/}(x_{2})]b_{1}^{\left(2\right)}$$

(41) $$+i\xi_{2}\sum_{m=0}^{\infty }(m+1)\xi_{1}^{m+1}a_{m}^{\left(1\right)}+3\sqrt{2}i\tau_{2}[j_{1}(x_{2})-x_{2}j_{1}^{/}(x_{2})]\sum_{m=1}^{\infty }\kappa_{1m}^{\left(1\right)}b_{m}^{\left(1\right)}=0,$$

and for n≥2,

$$\frac{\omega_{1n}^{2}R_{10}}{(n+1)\omega }s_{n}^{\left(1\right)}=[(n+1)(n+2)\tau_{1}-i]a_{n}^{\left(1\right)}+n(n+1)\tau_{1}[h_{n}^{\left(1\right)}(x_{1})-x_{1}h_{n}^{(1)/}(x_{1})]b_{n}^{\left(1\right)}$$

(42) $$+[n(n-1)\tau_{1}-i]\xi_{1}^{n}\sum_{m=0}^{\infty }{\left(-1\right)}^{m}C_{nm}\xi_{2}^{m+1}a_{m}^{\left(2\right)}+\tau_{1}i^{n}(2n+1)\sqrt{n(n+1)}[j_{n}(x_{1})-x_{1}j_{n}^{/}(x_{1})]\sum_{m=1}^{\infty }\kappa_{nm}^{\left(2\right)}b_{m}^{\left(2\right)},$$

$$\frac{\omega_{2n}^{2}R_{20}}{(n+1)\omega }s_{n}^{\left(2\right)}=[(n+1)(n+2)\tau_{2}-i]a_{n}^{\left(2\right)}+n(n+1)\tau_{2}[h_{n}^{\left(1\right)}(x_{2})-x_{2}h_{n}^{(1)/}(x_{2})]b_{n}^{\left(2\right)}$$

(43) $$+{\left(-1\right)}^{n}[n(n-1)\tau_{2}-i]\xi_{2}^{n}\sum_{m=0}^{\infty }C_{nm}\xi_{1}^{m+1}a_{m}^{\left(1\right)}+\tau_{2}i^{n}(2n+1)\sqrt{n(n+1)}[j_{n}(x_{2})-x_{2}j_{n}^{/}(x_{2})]\sum_{m=1}^{\infty }\kappa_{nm}^{\left(1\right)}b_{m}^{\left(1\right)},$$

where $x_{j}=k_{v}R_{j0}$,

(44) $$\omega_{j0}=\frac{1}{R_{j0}}\sqrt{\frac{1}{\rho }\left(3\gamma P_{gj}-\frac{2\sigma }{R_{j0}}\right)}$$

is the natural frequency of the radial mode of the *j*th bubble,

(45) $$\omega_{jn}=\sqrt{(n^{2}-1)(n+2)\frac{\sigma }{\rho R_{j0}^{3}}}$$

is the natural frequency of the *n*th shape mode of the *j*th bubble (n≥2),

(46) $$\tau_{j}=\frac{2\nu }{\omega R_{j0}^{2}},$$

(47) $$\kappa_{nm}^{\left(j\right)}=\frac{\sqrt{m(m+1)}}{(2m+1)i^{m}}\sum_{l=0}^{\infty }{\left(-1\right)}^{jl}i^{-l}(2l+1)C_{n0l0}^{m0}C_{n1l0}^{m1}h_{l}^{\left(1\right)}(k_{v}d).$$

It also follows from Equation (31) that

(48) $$P_{gj}=P_{0}+\frac{2\sigma }{R_{j0}}.$$

To make the system of Equations (38)–(43) closed, we use Equations (A19)–(A38) of [19], which were obtained there from the boundary conditions for the normal component of the liquid velocity and the liquid tangential stress. The above equations give for n=0,

(49) $$a_{0}^{\left(1\right)}=i\omega R_{10}s_{0}^{\left(1\right)},$$

(50) $$a_{0}^{\left(2\right)}=i\omega R_{20}s_{0}^{\left(2\right)},$$

and for n≥1,

(51) $$a_{n}^{\left(1\right)}+f_{n}^{\left(1\right)}b_{n}^{\left(1\right)}-n\sum_{m=0}^{\infty }\alpha_{2nm}a_{m}^{\left(2\right)}+\sum_{m=1}^{\infty }\beta_{2nm}b_{m}^{\left(2\right)}=\frac{i\omega R_{10}}{n+1}s_{n}^{\left(1\right)},$$

(52) $$a_{n}^{\left(2\right)}+f_{n}^{\left(2\right)}b_{n}^{\left(2\right)}-n\sum_{m=0}^{\infty }\alpha_{1nm}a_{m}^{\left(1\right)}+\sum_{m=1}^{\infty }\beta_{1nm}b_{m}^{\left(1\right)}=\frac{i\omega R_{20}}{n+1}s_{n}^{\left(2\right)},$$

(53) $$a_{n}^{\left(1\right)}+g_{n}^{\left(1\right)}b_{n}^{\left(1\right)}-\frac{n^{2}-1}{n+2}\sum_{m=0}^{\infty }\alpha_{2nm}a_{m}^{\left(2\right)}+\sum_{m=1}^{\infty }\gamma_{2nm}b_{m}^{\left(2\right)}=0,$$

(54) $$a_{n}^{\left(2\right)}+g_{n}^{\left(2\right)}b_{n}^{\left(2\right)}-\frac{n^{2}-1}{n+2}\sum_{m=0}^{\infty }\alpha_{1nm}a_{m}^{\left(1\right)}+\sum_{m=1}^{\infty }\gamma_{1nm}b_{m}^{\left(1\right)}=0,$$

where the coefficients $f_{n}^{\left(j\right)}$, $g_{n}^{\left(j\right)}$, $\alpha_{jnm}$, $\beta_{jnm}$ and $\gamma_{jnm}$ are calculated by Equations (A25)–(A34) of [19].

Combining Equations (38), (39), (49) and (50), one obtains

(55) $$\left(1-\frac{\omega_{10}^{2}}{\omega^{2}}+2i\tau_{1}\right)a_{0}^{\left(1\right)}+\sum_{m=0}^{\infty }{\left(-1\right)}^{m}\xi_{2}^{m+1}a_{m}^{\left(2\right)}=\frac{iP_{a}}{\rho \omega },$$

(56) $$\left(1-\frac{\omega_{20}^{2}}{\omega^{2}}+2i\tau_{2}\right)a_{0}^{\left(2\right)}+\sum_{m=0}^{\infty }\xi_{1}^{m+1}a_{m}^{\left(1\right)}=\frac{iP_{a}}{\rho \omega }.$$

Combining Equations (42), (43), (51) and (52), one obtains for n≥2,

$$\left[1-\frac{\omega_{1n}^{2}}{\omega^{2}}+i(n+1)(n+2)\tau_{1}\right]a_{n}^{\left(1\right)}+\left\{in(n+1)\tau_{1}\left[h_{n}^{\left(1\right)}(x_{1})-x_{1}h_{n}^{(1)/}(x_{1})\right]-\frac{\omega_{1n}^{2}}{\omega^{2}}f_{n}^{\left(1\right)}\right\}b_{n}^{\left(1\right)}\;+\sum_{m=0}^{\infty }\left\{{\left(-1\right)}^{m}C_{nm}\left[n(n-1)i\tau_{1}+1\right]\xi_{1}^{n}\xi_{2}^{m+1}+n\frac{\omega_{1n}^{2}}{\omega^{2}}\alpha_{2nm}\right\}a_{m}^{\left(2\right)}$$

(57) $$+\sum_{m=1}^{\infty }\left\{\tau_{1}i^{n+1}(2n+1)\sqrt{n(n+1)}\left[j_{n}(x_{1})-x_{1}j_{n}^{/}(x_{1})\right]\kappa_{nm}^{\left(2\right)}-\frac{\omega_{1n}^{2}}{\omega^{2}}\beta_{2nm}\right\}b_{m}^{\left(2\right)}=0,$$

$$\left[1-\frac{\omega_{2n}^{2}}{\omega^{2}}+i(n+1)(n+2)\tau_{2}\right]a_{n}^{\left(2\right)}+\left\{in(n+1)\tau_{2}\left[h_{n}^{\left(1\right)}(x_{2})-x_{2}h_{n}^{(1)/}(x_{2})\right]-\frac{\omega_{2n}^{2}}{\omega^{2}}f_{n}^{\left(2\right)}\right\}b_{n}^{\left(2\right)}\;+\sum_{m=0}^{\infty }\left\{{\left(-1\right)}^{n}C_{nm}\left[n(n-1)i\tau_{2}+1\right]\xi_{2}^{n}\xi_{1}^{m+1}+n\frac{\omega_{2n}^{2}}{\omega^{2}}\alpha_{1nm}\right\}a_{m}^{\left(1\right)}$$

(58) $$+\sum_{m=1}^{\infty }\left\{\tau_{2}i^{n+1}(2n+1)\sqrt{n(n+1)}\left[j_{n}(x_{2})-x_{2}j_{n}^{/}(x_{2})\right]\kappa_{nm}^{\left(1\right)}-\frac{\omega_{2n}^{2}}{\omega^{2}}\beta_{1nm}\right\}b_{m}^{\left(1\right)}=0.$$

Equations (40), (41), (53), and (54) do not contain the mode amplitudes $s_{n}^{\left(j\right)}$ and therefore remain unchanged.

Equations (40), (41), (53), (54) and (55)–(58) form a system of equations in the unknowns $a_{n}^{\left(j\right)}$ and $b_{n}^{\left(j\right)}$. The number of the equations, just as the number of the coefficients $a_{n}^{\left(j\right)}$ and $b_{n}^{\left(j\right)}$, is infinite. However, since $a_{n}^{\left(j\right)}$ and $b_{n}^{\left(j\right)}$ decrease for n→∞, their number can be truncated at some value of n=N. Doing so, we obtain a finite system of 4N+2 equations, which can be solved numerically,

(59) $$\left(1-\frac{\omega_{10}^{2}}{\omega^{2}}+2i\tau_{1}\right)a_{0}^{\left(1\right)}+\sum_{n=0}^{N}{\left(-1\right)}^{n}\xi_{2}^{n+1}a_{n}^{\left(2\right)}=\frac{iP_{a}}{\rho \omega },$$

(60) $$\sum_{n=0}^{N}\xi_{1}^{n+1}a_{n}^{\left(1\right)}+\left(1-\frac{\omega_{20}^{2}}{\omega^{2}}+2i\tau_{2}\right)a_{0}^{\left(2\right)}=\frac{iP_{a}}{\rho \omega },$$

$$(6\tau_{1}-i)a_{1}^{\left(1\right)}-i\xi_{1}\sum_{m=0}^{N}{\left(-1\right)}^{m}(m+1)\xi_{2}^{m+1}a_{m}^{\left(2\right)}$$

(61) $$+2\tau_{1}[h_{1}^{\left(1\right)}(x_{1})-x_{1}h_{1}^{(1)/}(x_{1})]b_{1}^{\left(1\right)}+3\sqrt{2}i\tau_{1}[j_{1}(x_{1})-x_{1}j_{1}^{/}(x_{1})]\sum_{m=1}^{N}\kappa_{1m}^{\left(2\right)}b_{m}^{\left(2\right)}=0,$$

$$i\xi_{2}\sum_{m=0}^{N}(m+1)\xi_{1}^{m+1}a_{m}^{\left(1\right)}+(6\tau_{2}-i)a_{1}^{\left(2\right)}$$

(62) $$+3\sqrt{2}i\tau_{2}[j_{1}(x_{2})-x_{2}j_{1}^{/}(x_{2})]\sum_{m=1}^{N}\kappa_{1m}^{\left(1\right)}b_{m}^{\left(1\right)}+2\tau_{2}[h_{1}^{\left(1\right)}(x_{2})-x_{2}h_{1}^{(1)/}(x_{2})]b_{1}^{\left(2\right)}=0,$$

$$\left[1-\frac{\omega_{1n}^{2}}{\omega^{2}}+i(n+1)(n+2)\tau_{1}\right]a_{n}^{\left(1\right)}+\sum_{m=0}^{N}\left\{{\left(-1\right)}^{m}C_{nm}\left[n(n-1)i\tau_{1}+1\right]\xi_{1}^{n}\xi_{2}^{m+1}+n\frac{\omega_{1n}^{2}}{\omega^{2}}\alpha_{2nm}\right\}a_{m}^{\left(2\right)}\;+\left\{in(n+1)\tau_{1}\left[h_{n}^{\left(1\right)}(x_{1})-x_{1}h_{n}^{(1)/}(x_{1})\right]-\frac{\omega_{1n}^{2}}{\omega^{2}}f_{n}^{\left(1\right)}\right\}b_{n}^{\left(1\right)}$$

(63) $$+\sum_{m=1}^{N}\left\{\tau_{1}i^{n+1}(2n+1)\sqrt{n(n+1)}\left[j_{n}(x_{1})-x_{1}j_{n}^{/}(x_{1})\right]\kappa_{nm}^{\left(2\right)}-\frac{\omega_{1n}^{2}}{\omega^{2}}\beta_{2nm}\right\}b_{m}^{\left(2\right)}=0,\;2\leq n\leq N,$$

$$\sum_{m=0}^{N}\left\{{\left(-1\right)}^{n}C_{nm}\left[n(n-1)i\tau_{2}+1\right]\xi_{2}^{n}\xi_{1}^{m+1}+n\frac{\omega_{2n}^{2}}{\omega^{2}}\alpha_{1nm}\right\}a_{m}^{\left(1\right)}+\left[1-\frac{\omega_{2n}^{2}}{\omega^{2}}+i(n+1)(n+2)\tau_{2}\right]a_{n}^{\left(2\right)}\;+\sum_{m=1}^{N}\left\{\tau_{2}i^{n+1}(2n+1)\sqrt{n(n+1)}\left[j_{n}(x_{2})-x_{2}j_{n}^{/}(x_{2})\right]\kappa_{nm}^{\left(1\right)}-\frac{\omega_{2n}^{2}}{\omega^{2}}\beta_{1nm}\right\}b_{m}^{\left(1\right)}$$

(64) $$+\left\{in(n+1)\tau_{2}\left[h_{n}^{\left(1\right)}(x_{2})-x_{2}h_{n}^{(1)/}(x_{2})\right]-\frac{\omega_{2n}^{2}}{\omega^{2}}f_{n}^{\left(2\right)}\right\}b_{n}^{\left(2\right)}=0,\;2\leq n\leq N,$$

(65) $$a_{n}^{\left(1\right)}+\frac{1-n^{2}}{n+2}\sum_{m=0}^{N}\alpha_{2nm}a_{m}^{\left(2\right)}+g_{n}^{\left(1\right)}b_{n}^{\left(1\right)}+\sum_{m=1}^{N}\gamma_{2nm}b_{m}^{\left(2\right)}=0,1\leq n\leq N,$$

(66) $$\frac{1-n^{2}}{n+2}\sum_{m=0}^{N}\alpha_{1nm}a_{m}^{\left(1\right)}+a_{n}^{\left(2\right)}+\sum_{m=1}^{N}\gamma_{1nm}b_{m}^{\left(1\right)}+g_{n}^{\left(2\right)}b_{n}^{\left(2\right)}=0,1\leq n\leq N.$$

Equations (51) and (52) at n=1 are not used when $a_{n}^{\left(j\right)}$ and $b_{n}^{\left(j\right)}$ are calculated. They define the amplitudes of the translational oscillations of the bubbles, $s_{1}^{\left(1\right)}$ and $s_{1}^{\left(2\right)}$.

Changing N in Equations (59)–(66), one can calculate the linear scattering coefficients $a_{n}^{\left(j\right)}$ and $b_{n}^{\left(j\right)}$ with any desired accuracy.

### 2.3. Net Force Experienced by Two Contacting Bubbles

The theory developed above allows one to calculate the forces on the bubbles, $F_{1}$ and $F_{2}$, at any distance d between the bubbles. For the bubbles in contact, we should set $d=R_{10}+R_{20}$. The problem is that at this value of d division by zero appears in equations obtained in [19] for acoustic microstreaming, which are used in the present paper. To overcome this problem, the forces on the bubbles can be calculated at a very small but nonzero distance between the bubbles’ surfaces and then the sum $F_{1}+F_{2}$ can be used as approximation of the net force acting on the two bubbles in contact. It is reasonable to assume that this approach should provide the net force on two contacting bubbles with acceptable accuracy.

## 3. Numerical Simulations

We consider the case without parametric excitation and calculate the linear scattering coefficients by means of the equations derived in Section 2.2. The following material parameters are used: ρ=1000 kg/m^3^, η=0.001 Pa s, σ=0.0727 N/m, γ=1.4, $P_{0}=101.3$ kPa, $P_{a}=10$ kPa, f=ω/2π=30 kHz. These parameters correspond to air bubbles in water.

**Figure 2:**
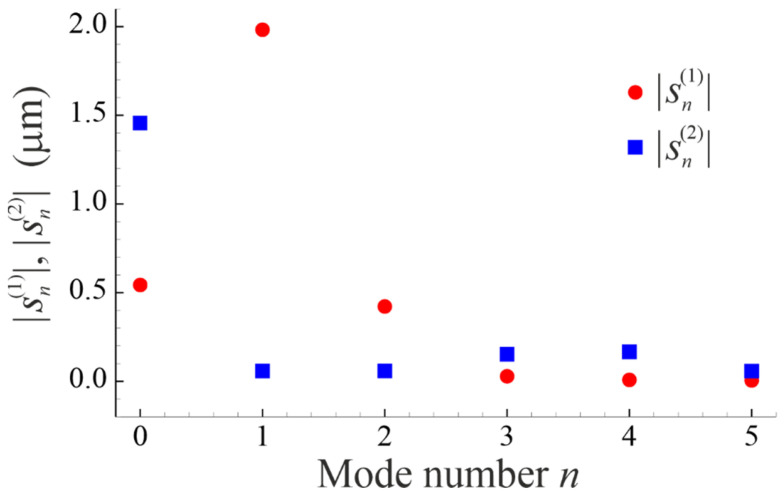
The magnitudes of the mode amplitudes $s_{n}^{\left(j\right)}$ for a bubbleman with $R_{10}=20$ μm and $R_{20}=50$ μm in the case that parametric excitation is absent. The calculation was carried out by the equations derived in Section 2.2.

When parametric excitation is absent, dominant oscillation modes are the radial mode (mode 0) and the translational mode (mode 1). As an example, Figure 2 shows the magnitudes of the mode amplitudes $s_{n}^{\left(j\right)}$ for two contacting bubbles (bubbleman) with $R_{10}=20$ μm and $R_{20}=50$ μm. As one can see, for bubble 1 (smaller bubble), the translational mode is dominant, while for bubble 2 (bigger bubble), the radial mode is dominant.

**Figure 3:**
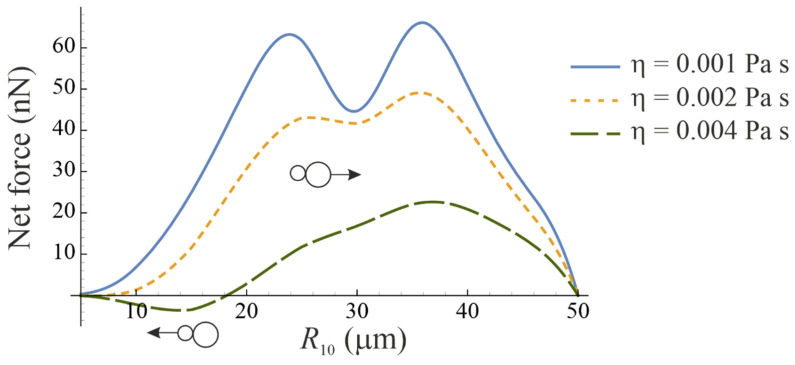
Net force experienced by a bubbleman in the case that parametric excitation is absent. The equilibrium radius of bubble 2 is kept fixed, $R_{20}=50$ μm, while the equilibrium radius of bubble 1 is varied.

Figure 3 shows the net force experienced by the bubbleman. The equilibrium radius of bubble 2 is kept fixed, $R_{20}=50$ μm, while the equilibrium radius of bubble 1 is varied. The calculation was made for three values of the liquid viscosity η. As one can see, the force magnitude decreases with increasing viscosity. For lower viscosity, the net force is always directed from the smaller bubble to the bigger bubble, while for higher viscosity, the force can act in the opposite direction. As expected, the net force vanishes for $R_{10}=R_{20}$ and for $R_{10}\to 0$.

**Figure 4:**
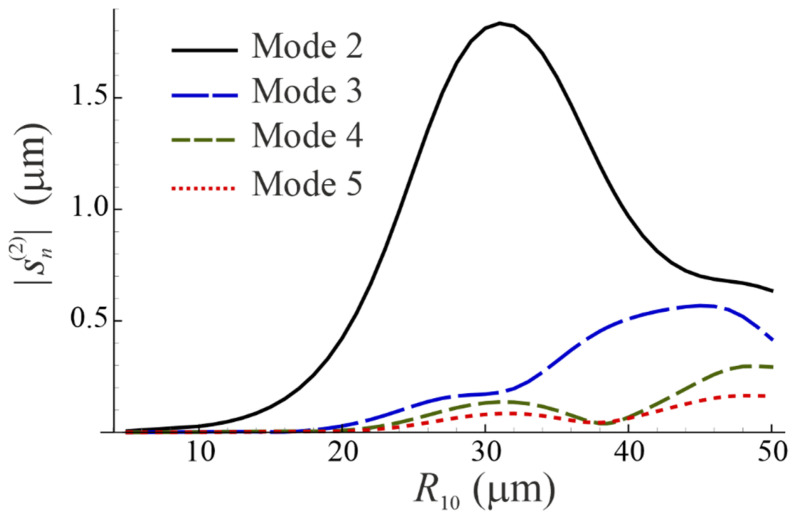
The magnitude of the shape modes developing on bubble 1 as a function of $R_{10}$. The equilibrium radius of bubble 2 is kept fixed, $R_{20}=50$ μm, η=0.001 Pa s, and the other parameters are as in Figure 3.

Figure 4 shows the magnitude of the shape modes developing on bubble 1 as a function of $R_{10}$. The equilibrium radius of bubble 2 is kept fixed, $R_{20}=50$ μm, η=0.001 Pa s, and the other parameters are as in Figure 3. The comparison of Figure 3 and Figure 4 suggests that the trough in the upper curve of Figure 3 is caused by the resonance of mode 2 of bubble 1, as at $R_{10}=29.065$ μm, the driving frequency of 30 kHz is equal to the natural frequency of the second shape mode; see Equation (45).

**Figure 5:**
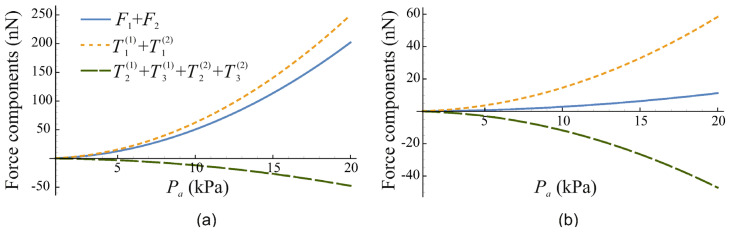
The contributions of the force components, given by Equations (14), (15) and (17), to the net force $F_{1}+F_{2}$ on the bubbleman as a function of the acoustic pressure amplitude $P_{a}$ for (**a**) η=0.001 Pa s and (**b**) η=0.004 Pa s. $R_{10}=20$ μm, $R_{20}=50$ μm, f=30 kHz.

Figure 5 shows the contributions of the force components, given by Equations (14), (15) and (17), to the net force on the bubbleman as a function of the acoustic pressure amplitude $P_{a}$ for two values of the liquid viscosity, η=0.001 Pa s and η=0.004 Pa s. It is assumed that $R_{10}=20$ μm, $R_{20}=50$ μm and f=30 kHz. The solid curve shows the net force $F_{1}+F_{2}$, the short-dash curve shows the contribution of the terms $T_{1}^{\left(j\right)}$ given by Equation (14), and the long-dash curve shows the contribution of the terms $T_{2}^{\left(j\right)}$ and $T_{3}^{\left(j\right)}$ given by Equations (15) and (17). As one can see, at η=0.001 Pa s, the dominant contribution comes from the terms $T_{1}^{\left(j\right)}$, while the terms $T_{2}^{\left(j\right)}$ and $T_{3}^{\left(j\right)}$, which arise due to acoustic microstreaming, reduce the net force slightly. At η=0.004 Pa s, the contribution of the terms $T_{2}^{\left(j\right)}$ and $T_{3}^{\left(j\right)}$ becomes comparable to the contribution of the terms $T_{1}^{\left(j\right)}$, which results in a considerable reduction in the net force.

## 4. Experiments

**Figure 6:**
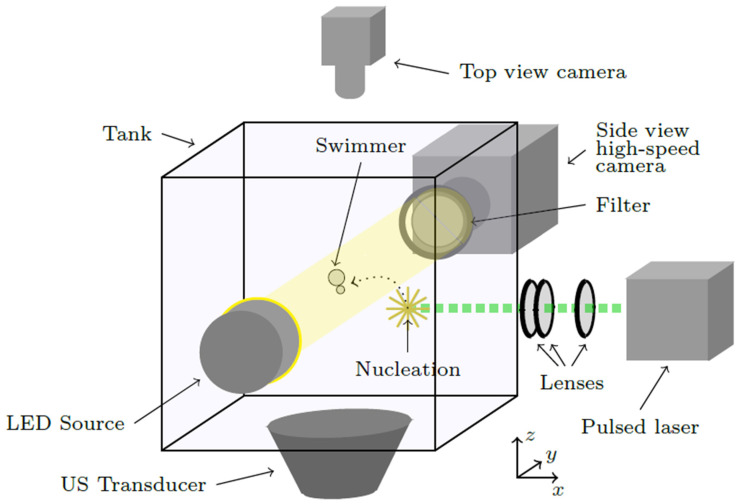
Schematic of the experimental setup used for the creation of a two-bubble microswimmer (bubbleman) and for the capture of its oscillation and translation dynamics.

The experimental design of the proposed microswimmer requires controlling the approach and the contact of two gas bubbles in an infinite medium. To this end, we take advantage of an experimental setup that allows one to control the coalescence of two bubbles in an acoustic levitation chamber. The setup and the experimental technique have already been described in detail in one of our previous studies [29], so here we only describe shortly the main experimental activities. Figure 6 depicts the experimental setup that is used for the creation of a two-bubble microswimmer (bubbleman) as well as for the capture of its oscillation dynamics and its translational trajectory in two orthogonal planes.

An 8 cm-edge cubic tank is filled with a viscous fluid medium constituted of sodium alginate power mixed with water (at a concentration of 2 g/L), whose viscosity has been measured by using a Anton Paar rheometer (MCR302 equipped with parallel plate) at 4.3 mPa s. Single bubbles are nucleated by short laser pulses using a Nd: YAG pulsed laser (λ=532 nm New Wave Solo PIVIII), focused by a lens set. This laser-nucleation system allows generating microbubbles with a radius ranging from 20 µm to 50 µm. A 30.8-kHz Langevin transducer, which is in contact (by means of an echographic gel) with the underside of the tank, generates an acoustic standing wave field inside the tank, the driving frequency of which corresponds to a certain acoustic resonant mode of the cubic cavity. The standing wave has at least one pressure antinode that is located approximately at the middle-top part of the tank. This pressure antinode is a stable equilibrium location for bubbles with radii smaller than the resonance radius at the driving frequency, which is estimated by Equation (44) to be about 110 μm.

**Figure 7:**
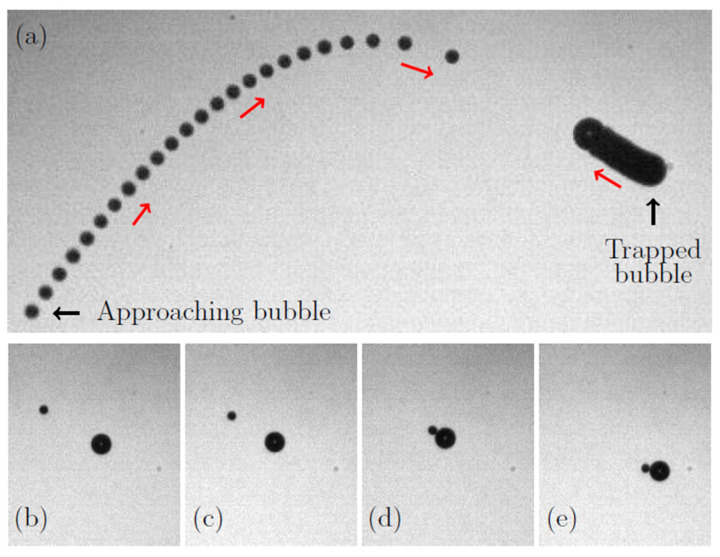
(**a**) Bubble trajectories prior to the contact. A first bubble (right side) is trapped at a stable location in the acoustic standing wave. Once a second bubble is nucleated (left side), it moves due to the primary radiation force towards the same stable location. When the bubbles come to proximity, the radiation interaction force makes them come into contact. (**b**,**c**) The last moments prior to the contact. (**d**) The bubbles come into contact and remain in this state. (**e**) As soon as the bubbles come into contact, the bubbleman moves to a new stable location.

The process of the creation of a bubbleman is illustrated by Figure 7. A first bubble is nucleated by the laser at a distance of a few millimeters from the pressure antinode. Due to the driving acoustic field, this bubble oscillates spherically and is subject to a primary radiation force that makes it move to the nearest pressure antinode and settle there; see the trapped bubble on the right side of Figure 7a.

A second bubble is then nucleated; see the bubble on the left side of Figure 7a. The trajectory of this bubble is first determined by the primary radiation force and then by the secondary radiation (interaction) force that acts between the two bubbles when they are in close proximity. The interaction force exerted on the first (right) trapped bubble results in a small translational motion towards the approaching (left) bubble, as one can see in Figure 7a. At the final stage of the bubble approach, they can either coalesce to form a bigger bubble or come into contact and remain in this state. The exact mechanism underlying the coalescence and the contact of two bubbles is not yet well understood. The transition from coalescence to contacting bubbles surely depends on the velocities of approach of the bubbles, the amplitude of their spherical oscillations their equilibrium radii and their surface contamination. Anyway, although coalescence can happen in the levitation chamber, its absence is regularly observed and contacting bubble pairs often occur. Figure 7b–e show the last steps of the approach and the contact of two bubbles, which result in the creation of a bubbleman. Once the bubbleman is created (see Figure 7d), it moves slowly to a new trapping location, shown in Figure 7e, which results from a balance between the primary radiation force on the bubbleman and the buoyancy force.

**Figure 8:**
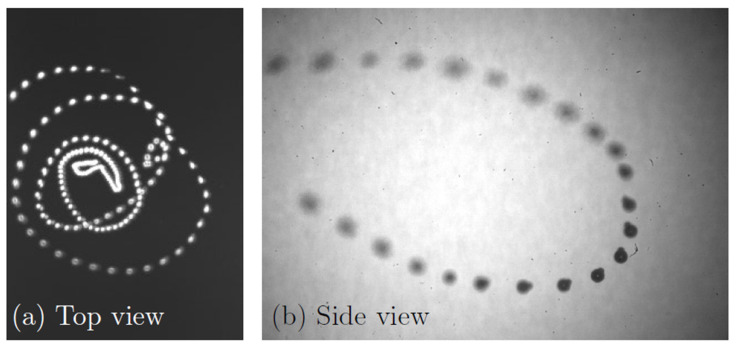
(**a**) Trajectory of the bubbleman captured from above. The diameter of the quasi-circular trajectory can reach 4 mm. (**b**) A zoom of the trajectory captured from the side. The bubbleman is clearly visible, showing that both bubbles remain spherical during the motion.

Since the bubbleman is located at a pressure antinode, both bubbles continuously undergo spherical oscillations and experience an interaction force, which is the source of a net propulsion force. However, if the interaction force does not exceed the trapping force caused by the primary radiation force, the bubbleman remains trapped. When the applied acoustic pressure is increased, the interaction force also increases and the net propulsion force can exceed an energy barrier that provides the trap of the contacting bubbles. As a result, the bubbleman begins to move and leaves the pressure antinode. Figure 8 shows the trajectory of the bubbleman captured from two orthogonal views. One camera (Phantom v12.1 equipped with a 12× Navitar objective lens, Vision Research, USA) captures the motion of the bubbleman from the side. A frame size of 1280 × 800 pixels (magnification of 4.6 µm/px) is used at a 60-Hz frame rate. Backlight illumination is provided by a continuous light-emitted diode (LED) light source. This allows capturing with high accuracy the shape of the bubbleman and ensures that it is spherically oscillating; see Figure 8b. Another camera (Basler ac640–750 µm) captures the top view of the bubbleman motion with a 640 × 480 frame size (magnification of 22 µm/px) at a 60-Hz frame rate. Due to experimental constraints, it is not possible to used backlight for the top view observation. The bubbleman is visualized thanks to the scattered light from the LED light source. The exposure time of the camera is therefore increased significantly in order to capture a bright spot corresponding to the propeller; see Figure 8a. The bubbleman under study is constituted of two bubbles with equilibrium radii of $R_{10}=60$ μm and $R_{20}=25$ μm. The propeller exhibits quasi-circular orbits around the pressure antinode with a predominant motion in the plane orthogonal to the direction of gravity. As one can see from the side view, the largest travelled distance is ~1 mm. From the top view, the diameter of the largest orbit is around 4 mm. The propeller velocity has been estimated to reach 0.8 cm/s, which corresponds to 47 body lengths per second. The motion of the bubbleman can last as long as 10 s. For an acoustic frequency of 30.8 kHz, this corresponds to a bubbleman performing spherical oscillations for more than 300,000 acoustic periods. It is worth noting that such velocities correspond to the fastest acoustically-powered microswimmers mentioned in the literature. At the end of the motion, the intensity of the propulsion force decreases and the bubbleman becomes trapped again, returning to its equilibrium location. This loss of propulsion efficiency probably comes from a change in the bubble equilibrium radii due to rectified diffusion, a phenomenon that occurs on long timescales. Such a size modification can change drastically the magnitude of the net propulsion force, resulting then in a lack of motion. This effect can be prevented by coating bubble surfaces, which should enhance the size stability of the bubbles. At this stage, we cannot easily compare the experimental trajectories to the theoretical modelling. The main reason is that the theoretical modelling allows the calculation of the net propulsion force but not of the motion of the system within the levitation chamber. In order to describe the trajectories of the bubbleman, it is necessary to derive the equation of motion of the two-bubble system in the three-dimensional acoustic field by including the forces acting on the system: the buoyancy, the drag force, the radiation interaction force, and the primary radiation force (induced by the ultrasound field onto the bubbleman itself). This requires knowledge of the 3D pressure field around the trapping location, and relevant experiments are currently in progress in order to quantitatively determine the pressure field in the levitation chamber and hence to predict and control the trajectory of the bubbleman.

## 5. Conclusions

In this paper, a theory has been developed that allows one to analytically calculate the acoustic radiation interaction forces between two gas bubbles in an incompressible viscous liquid for any small separation distance between the bubbles. This theory has been used to demonstrate that two acoustically excited bubbles experience a nonzero net force when they come into contact. This result suggests a new mechanism that can be used for the development of artificial self-propelled microswimmers actuated and controlled by an acoustic field.

## Data Availability

The data that support the findings of this study are available from the corresponding author upon reasonable request.

## Appendix A. Mathematical Formulas Used in Calculations

Here, we provide auxiliary mathematical formulas that are used in our derivation [23],

(A1) $$\int_{-1}^{1}P_{n}(\mu )P_{m}(\mu )d\mu =\frac{2}{2n+1}\delta_{nm},$$

(A2) $$\mu P_{n}(\mu )=\frac{n}{2n+1}P_{n-1}(\mu )+\frac{n+1}{2n+1}P_{n+1}(\mu ),$$

(A3) $$\sqrt{1-\mu^{2}}P_{n}^{1}(\mu )=\frac{n(n+1)}{2n+1}\left[P_{n+1}(\mu )-P_{n-1}(\mu )\right],$$

(A4) $$\int_{-1}^{1}P_{n}^{1}(\mu )P_{m}^{1}(\mu )d\mu =\frac{2n(n+1)}{2n+1}\delta_{nm},$$

where $\delta_{nm}$ is the Kronecker delta.
